# Exploring the relative importance of work-organizational burnout risk factors in Belgian residents

**DOI:** 10.1080/10872981.2018.1521246

**Published:** 2018-09-23

**Authors:** Ruben Willems, Chris Monten, Gwendolyn Portzky

**Affiliations:** a Department of Public Health, Ghent University, Ghent, Belgium; b Department of Radiation Oncology and Experimental Cancer Research, Ghent University, Ghent, Belgium; c Department of Psychiatry and Medical Psychology, Ghent University, Ghent, Belgium

**Keywords:** Burnout, residents, work-organizational factors, education, stress

## Abstract

Previous research has shown that residents are at risk for developing burnout. Most burnout measures focus on individual risk factors, although work-organizational-focused measures might be beneficial as well. This study analyzed the relative importance of positive and negative work-organizational stressors, according to residents themselves, with NVivo11. Eleven work-organizational themes were found with deductive reasoning and two themes, recognition and success experiences, were found inductively. Main positive stressors are professional development, receiving feedback, experiencing success, autonomy and social support. Main negative stressors are high workloads, role conflicts/ambiguity, long work hours, and a lack of feedback, a lack of social support, and a lack of professional development. Measures to improve residents’ well-being should not only focus on reducing workload and work hours. Our results suggest to allocate resources to improve supervisors’ skills, such as providing social support, feedback, and recognition. A better match between internship obligations and residents’ studies could also contribute positively to this purpose.

## Introduction

Residents are at the highest risk within the medical profession to develop burnout [1]. Previous research in our study population revealed that 8.5% of residents experienced burnout and that 27.8% were at risk [2]. Other research reported prevalence figures of burnout in residents varying between 18% and 82% [3].

Burnout is characterized by three specific though interrelated dimensions [4]. *Emotional exhaustion* refers to extreme fatigue and emotional depletion resulting from excessive job demands. *Depersonalization* is a negative, cynical, and disconnected attitude toward work and includes psychological withdrawal from work. It is a self-protecting mechanism against exhaustion and disappointment. *Reduced personal accomplishment* is the third dimension of burnout [4], referring to a negative perception of personal competences and job functioning. Research validated the contribution of these three dimensions across cultures, countries, languages, and professions [5]. Burnout may influence the person’s overall physical and psychological well-being [6,7] resulting in health-care organizations facing higher rates of absenteeism [8], job turnover [9], and suboptimal medical care [10,11].

The majority of burnout intervention studies in health-care populations focus on individual risk factors [12]. Research on burnout measures in residents had a more balanced focus between individual and work-organizational measures [1,13–16] but burnout was seldom used as a dependent variable. Only a small correlation was found between individual factors and burnout in Prins et al.’s review [3]. Furthermore, both Awa et al. [12] and Maslach et al. [17] suggested that work-organizational measures are indispensable in reducing burnout.

### Study purpose

The current study aims to explore the relative importance of positive and negative work-organizational stressors in burnout. The identified risk factors will be compared to the predicted risk factors. Identified stressors could guide further research and direct management in taking effective burnout prevention measures on the work floor.

## Method

### Procedure and participants

Residents associated with Ghent University, Belgium, are asked every year to fill in a training-satisfaction survey. Ghent University offers over 30 medical specialist trainings. All were found eligible to include in our study. Belgian residents may work 48 h a week on average with a maximum of 60 h a week. Shifts cannot exceed a consecutive 24 h and a 12-h break is obligated after a shift of at least 12 h [18]. However, longer workweeks (55–60 h a week) than legally defined were found in previous research in our study population. Compared to specialists, they also have more night and weekend shifts [2].

This retrospective research analyzed data from the 2015 and 2016 training-satisfaction surveys. The 2015 and 2016 survey period lasted 5 and 7 weeks, respectively. Residents were invited to participate in the survey by email. Respectively, four and eight reminders were sent to encourage participation. A total of 236 (37.5%) in 2015 and 311 (47.4%) residents filled in the questionnaire. This analysis is limited to the two open-ended questions on work-organizational stressors, presented in both satisfaction surveys:

1. What is a positive stressor for you as a resident?
2. What is a negative stressor for you as a resident?

The response rate on the first question regarding positive stressors increased from 19.1% in 2015 to 30.5% in 2016. A similar increase was noticed for the question regarding negative stressors, from 20.7% to 31.3%, respectively.

### Analysis

Data have been analyzed with NVivo 11 (NVivo qualitative data analysis Software; QSR International Pty Ltd. Version 11, 2016) by the first author (RW). We followed the guidelines of Braun & Clarke [19] in handling the data. The first author analyzed the data iteratively to overcome difficulties. An analyst-driven thematic analysis was conducted, based on an a priori scoping review of the literature to identify potential burnout risk factors (Table 1). Three risk categories were identified and considered to be the main themes: (a) individual factors, (b) job demands, and (c) a lack of job resources. The latter two form the work-organizational risk factors. All identified risk factors were categorized under one of the main themes. The deductive derived subthemes did not capture all data. Hence, data-driven subthemes were defined if needed.

**Table 1.** In literature described potential risk factors.

Individual Factors	Job Demands	Job Resources
Age	Workload	Autonomy
Gender	Work time	Participation
Personality factors	Role conflicts	Professional development
Job attitude	Role ambiguity	Feedback
Coping strategy	Emotional pressure	Social support
Marital state		Organizational culture
Physical activity		Communication and
Education level		Collaboration

Contrary to most other studies using NVivo analysis software, we chose to report only a few quotes to support our claims because the aim of this study is to investigate the relative importance of the reported risk and protective factors. Therefore, frequency analysis was performed with the Matrix Coding Query function.

### Institutional review board statement

Not every research plan requires ethics approval of an Ethics Commission at Ghent University. The researchers are responsible to judge the necessity of approval in good faith. In this case, no approval of an Ethics Commission was demanded because the primary goal of the survey was to evaluate the quality of the specialists’ education. Residents participated freely. Later on, we acknowledged the potential scientific value of the survey, resulting in this paper.

## Results

Demographic information can be found in Table 2. Female/male ratio was approximately 2:1, corresponding to the gender distribution of residents in previous research at Ghent University Hospital [2]. Residents in internal medicine, pediatric medicine, gynecology, anesthesia, and surgery covered almost half of all respondents.

**Table 2.** Demographic data.

		Survey 2015 (%) *N* = 236	Survey 2016 (%) *N* = 311
Response rate open-ended questions	Positive sources of tension	19.1	30.5
	Negative sources of tension	20.7	31.3
Female		67.6	65.9
Residency year	1st	18.6	19.9
	2nd	21.8	16.4
	3rd	20.7	19.0
	4th	13.3	19.9
	5th	13.3	14.5
	6th	4.8	4.5
	Extra specialization year	0	0
	Doctoral year	7.4	5.8

Table 3 gives an overview of the relative importance of the identified themes. In total, two data-driven themes (success experiences and recognition) and 13 work-organizational themes in total were identified. Positive stressors were mainly associated with job resources. Contrary, both job demands and a lack of job resources could be seen as a negative stressor. Professional development, receiving timely feedback, experiencing success, autonomy, and social support were the main positive stressors. A high workload, role conflicts and role ambiguity, long work hours, and a lack of feedback, a lack of social support, and a lack of professional development were identified as main negative stressors.

**Table 3.** Distribution of themes across stressors and combined percentage.

	Positive stressor (%)	Negative stressor (%)
Job resources	**87.27**	**47.42**
Autonomy	9.03	1.75
Communication and Collaboration	2.74	3.58
Recognition	3.87	1.49
Feedback	15.49	14.57
Organizational culture	0.17	1.43
Participation	2.37	3.9
Professional development	32.31	11.61
Social support	6.87	9.08
Success experiences	14.43	0
Individual factors	**4.04**	**0.52**
Physical activity	0.09	0
Job attitude	3.95	0.52
Job demands	**8.69**	**52.06**
Work hours	0.38	9.54
Emotional burden	1.56	3.3
Role conflicts and ambiguity	1.82	13.24
Workload	4.93	25.98

### Thematic results: job resources

Residents performing surgical/technical procedures or taking medical decisions independently experience autonomy. However, they lack autonomy in managing their agenda. Residents view a strong hierarchical medical structure as an obstacle to take on a participating role.

*Some staff members are unreasonable in meetings, with the only purpose to hold on to their authority.

Participation as a positive stressor is strengthened by opportunities to actively assist in medical procedures or having a voice in meetings and policy decisions.

Feedback could be divided into two concepts: direct and general feedback. Direct feedback is the opportunity to receive qualitative feedback from a supervisor during practice. Direct feedback is a positive stressor, especially if the resident lacks experience. However, residents often report late, passive, or absence of feedback, when confronted with challenging cases. General feedback can be given on any occasion. Negative feedback, next to positive feedback, is well appreciated when given in a constructive manner. As a result, negative and avoidance of feedback are reported as negative stressors.

Residents want to develop a professional identity through intellectual stimulation by working on challenging cases. They adhere importance to developing new skills on the work floor or through self-education.

*To have the opportunity to read an article to broaden your knowledge on a certain pathology.

However, little time for self-education after working hours is often reported. Work schedules are not always complementary with obligated training courses, leading to double-booked agendas.

*When you feel it is necessary to leave on time due to classes or social obligations, but at the start of the day you already feel it will not be possible because it is too busy.

Lack of social support of supervisor and staff is perceived as an important negative stressor. Social support (e.g., compassion, frustration ventilation) of co-residents and colleagues is reported as a positive stressor. On the contrary, an isolated position, competition with co-residents as well as tension and conflicts on the workplace are reported as negative stressors.

Data-driven analyses led to two complementary factors. Success experiences are reported as a positive stressor. Such events can be of different origin: a successful clinical diagnosis, the mastering of an intervention or a difficult situation, making professional progression, performing lifesaving interventions, or a patient’s recovery.

*An urgent problem which is not easy to solve, but I know it is within my capacities to find the solution.

A second inductively found factor is recognition. Although recognition is closely related to feedback, a clear distinction can be made. Feedback is focused on the content of the job: e.g., an incorrect diagnosis or treatment. With the term ‘recognition’, we address the appreciation for the residents’ efforts and time invested. Recognition is not merely provided by the supervisor, but can also be expressed by, for example, the patient. Recognition by staff or supervisor is considered as a positive stressor, whereas absence of recognition is signaled as a negative stressor. The same can be applied on recognition from patients and their families, although to a lower degree.

*Despite the high work pressure, there are patients appreciating all your efforts.

### Thematic results: job demands

A minority of residents experience a high workload as a positive stressor, but most residents indicate a high workload as a negative stressor. Overload is defined as an accumulation of multitasking, especially in case of recurrent interruptions, stressful situations, too many patients, educational obligations, understaffing, and an administrative overload.

*A packed agenda to manage and meanwhile you receive hundreds of telephone calls about nothing, and you have to help out a colleague who cannot manage his agenda.

The weekly work hours needed to manage the high workload are perceived as long and irregular. The 24-hour shifts are exhausting in particular. Both weekly work hours and shift length are equally referred to as negative stressors.

*There is tension between my work and private life. As a mother, I often have the feeling I fall short on the job, as a mother at home, and as a spouse.

Emotional burden is reported in case no help can be offered to deteriorating patients or when patients and/or their relatives are unhappy with the treatment, although the best care possible was given.

Supervisors’ and staff’s high expectations combined with conducting medical acts unguided can result in role ambiguity, especially if guidance was expected. Feelings of uncertainty and anxiety to make errors are reported. Role conflicts may also arise in case of contradictive instructions by supervisors.

*As a starting resident, you just have to start working, without much guidance. You feel like a cheater.

*You have several supervisors, and all have an opinion that can differ about some aspects of the job. You can impossibly do good for everybody. You follow the advice of one supervisor, you get criticism of the other supervisor.

## Discussion

Qualitative analysis of a survey on medical residents revealed that professional development, workload, and feedback were identified as the three most often mentioned positive or negative stressors, within a total of 13 different work-organizational-related factors. Two of these themes (success experiences and recognition) were not found in the available literature and were extracted through induction.

Our results suggest a pivotal role of the supervisor in burnout prevention. Although residents appreciate a certain level of autonomy and responsibilities, autonomy turns into a strong negative stressor if these levels exceed their capacities, especially if guidance is lacking. We argue that it is important to practice a *Guided Autonomy*, which we define as ‘autonomy, based on the resident’s competence level and the accessibility of supervision’. Galam, Komly, Le Tourneur & Jund [20] proposed a roadmap to support professional development, similar to our concept of Guided Autonomy, in which qualitative supervision is necessary in case the resident faces challenging cases. It is not always clear if the call for guidance should come from the supervisor or from the resident himself. The possible gap between both could be closed by constructive feedback on the residents’ performance.

Regular general feedback may reduce role ambiguity in residents and may increase recognition and social support from supervisors [20]. Again, this may lead to more success experiences such as mastering a skill or overcoming challenging situations. We hypothesize that such improvement in feedback, together with Guided Autonomy, may result in lower burnout rates among medical residents.

As could be expected, a high workload, excessive administration, long and irregular work hours, and 24-hour shifts are negative stressors. Working-time restriction is mainly investigated in US residents. In most studies, burnout scores decreased significantly with reduced working-time [21–23]. Literature is inconclusive whether this decrease is a consequence of weekly working-time restrictions or a restriction in shift length. Block, Feldman, Yeh, & Desai [24] suggest the latter. Lower levels of workload may lead to improved care and less medical errors [25]. Residents often have to multitask, which may result in lower concentration levels and suboptimal efficiency [26,27]. As a result, protracted and rushed working-hours may result in inefficient or even contra-productive on-the-job training.

Our results suggest that professional development can take place within and outside the workplace, similar to Schaufeli et al. [28] In general, residents reported a desire to acquire knowledge and skills through on-the-job training, and through self-education. However, time for self-education is perceived as limited due to internship obligations. Similar to Msaouel et al. [29], our study population questioned the validity of the obligated courses.

### Limitations

Our study has a number of limitations. First, as in all voluntary surveys, a selection bias cannot be excluded. As we did not assess participants’ burnout scores, residents who experience burnout may be under- or overrepresented in our population. Second, although the open-ended questions aimed at measuring work-organizational stressors, some responses included individual stressors as well. Our focus on work-organizational stressors consequently does not capture the whole picture. Third, only the first author analyzed the data set. Finally, data collection based on ‘free text’ allows capturing of new information in comparison to pre-specified options; it does, however, complicate the unequivocal comprehension of answers. Because surveys on work floor problems among medical residents may produce sensitive information, maximal anonymity was guaranteed. However, this strategy precluded the possibility of clarifying in-depth interviews.

### Research recommendations

A prospective multicenter study to investigate the generalizability of our results to all Belgian residents would be interesting. Future research on the association between working-time, shift length, and burnout may clarify the optimal balance between acceptable exposure to the work floor and its educational role. The effect of supervisors’ guidance and supervisors’ leadership style on burnout needs special attention, focusing on ‘Guided Autonomy’ and how to give constructive feedback. The role of recognition is still to be explored. At last, individual factors are potentially compatible with work-organizational burnout prevention measures, targeting different factors.

### Implications for practice

It is important to foster residents because they are co-responsible for the quality of our health care. Our results suggest altering policy into decreasing the workload and work hours, to reallocate time and resources to facilitate training of supervisors, and to strive for complementarity between internship and education.

The role of the supervisor is pivotal in the training of residents. It is therefore necessary to reallocate resources toward more intensive and specific training of supervisors. Those resources need to be provided on a hospital level or higher. Hospital managers should discuss the needs and expectations of both residents and their supervisors, the medical specialists, to determine potential opportunities and barriers. Supervisors need to be(come) aware of the determinants of a positive, informative, and satisfying residency period, such as regular feedback, autonomy, and proximity. Developing a flexible, personalized pathway per resident could be a positive step in creating as much success experiences as possible. The authors are well-aware of the time pressure and high workload supervisors experience themselves. Extra time spending on the supervisors’ side, due to more training or more time allocated for feedback, might implicate financial losses or extended workweeks. Financial compensation may be necessary but more administrative support could also free up time. Improved clinical care may eventually lead to less errors and therefore savings. Also, the pressure on residents could be slightly relieved if hospital managers and medical university representatives have regular meetings to overlook the complementarity of the internship and the obligatory classes. The feedback of residents on their curriculum is of utmost importance in the process of training improvements.

## Applying Research to Practice

- It is recommended to reallocate more resources toward more intensive training of supervisors in order to decrease burnout prevalence among residents.
- Finding and monitoring the right balance between autonomy and guidance is pivotal to achieve a maximum of success experiences.
- Residents question the complementarity of the tough internship and obligatory classes. Universities have to keep in mind that too much learning moments and too little spare time may lead to suboptimal learning levels.
