# Supplementary material for: Exploring the relative importance of work-organizational burnout risk factors in Belgian residents
Source: Med Educ Online. 2018 Sep 23;23(1):1521246. doi: 10.1080/10872981.2018.1521246 (PMC6161591; doi:10.1080/10872981.2018.1521246)
Supplement: Supplemental Material [file ZMEO_A_1521246_SM9009.docx]

Supplementary material – scoping review of potential burnout risk factors in health care sector workers with a focus on residents

# Individual risk factors

## Age

Burnout occurs in all age categories. Older employees have problems to keep up with rapidly changing medical technologies. They are stuck in a routine, leading to low levels of self-cultivation [1, 2]. However, older employees developed strategies to cope with potential burnout risks. They also experience higher levels of personal accomplishment. Hence younger employees (under 40 years old) are at higher risk to develop burnout [3, 4]. The age of Belgian residents averaged at 28.4 years,[5] and only a few residents are above 34 [6].

## Gender

Research on the impact of gender on burnout in different populations, including residents, is inconclusive [7-12]. Personal accomplishment and depersonalization is higher in males, and women score higher on emotional exhaustion [13].

## Personality traits

There is a clear association between the Big Five personality traits (openness to experiences, agreeableness, conscientiousness, extraversion and neuroticism) and burnout.[7] High scores on conscientiousness, including perfectionism, and low scores on extraversion are burnout risk factors in particular[14, 15]. High levels of resilience and hardiness mitigate the risk to develop burnout[3, 7, 16]. At last, residents with a disorganized personality are at risk[17].

## Job attitude

If employees have great expectations of their job and if they set high targets which are difficult to reach, they will put a lot of effort in their job. As a result, they might face emotional exhaustion and depersonalization [3, 7]. These results are reproduced in a sample of residents. The drive to work as much as possible can lead to role conflicts, a major burnout risk factor [18]. Big expectations might cause big disappointments, leading to pessimism, and having a negative effect on emotional exhaustion, depersonalization and personal accomplishment [19].

## Coping strategy

An active coping strategy reduces the burnout risk, contrary to an avoiding or emotional coping strategy [3, 7, 20]. Residents often oppress negative feelings to conduct their job to the best of their efforts [21].

## Marital status

Singles are at risk to develop burnout, in contrast to married people who are less prone to develop burnout. Divorced people know high levels of emotional exhaustion, comparable to singles. On the other hand, divorces people tend to have higher levels of personal accomplishment and lower levels depersonalization, just like married people. Emotional conflicts and interpersonal problems at work are better coped with, by people having a family [22, 23]. Several publications confirmed these observations in residents. The support of a partner is important, and residents with children are less cynical and more human in social interactions [24-26]. However, other research did not find an association between marital status and burnout prevalence in residents [5, 12].

## Physical activity and general lifestyle

People who are physical active score lower on burnout measurements [27]. In addition, the general health status predicts emotional exhaustion [20]. No research has focused on the association between physical activity and burnout in residents to the author’s knowledge.

## Education level

Residents have a very high education level. Both very high and very low education levels can lead to emotional exhaustion [22]. Pragmatic, on the job training improves the personal accomplishment of employees [28]. This might be a protective factor for residents.

# Job demands

## Workload

Residents experience a high workload caused by suboptimal efficiency, the combination of working and studying, vague boundaries because they want to prove themselves [29], insecurity, high amounts of work, potential career opportunities in the near future [18], and excessive administration [30], making them vulnerable to develop burnout [10, 21, 31].

## Work hours

Irregular and long work hours may have a negative impact on the work-life balance, next to sleep deprivation and unhealthy eating patterns, eventually leading to burnout [25]. In residents, irregular rest periods is associated with burnout [5]. A multi-country review found that residents work 54 hours on average. One Belgian study reporting an average work week of 63 hours was included [5]. In most countries, federal regulations restrict shift length and work hours. However, non-compliance to the law is widespread [32].

## Role conflicts and role ambiguity

Role conflicts result from conflicting orders on how to practice the job. These conflicting orders can come from one or several individuals. Role conflicts can lead to emotional exhaustion and depersonalization [33]. Vaguely defined job content and job responsibilities can cause role ambiguity. It prevents employees from developing and reaching goals [34]. Role ambiguity leads to higher levels of depersonalization and emotional exhaustion, and lower levels of personal accomplishment [33].

## Emotional pressure

Health care sector workers regularly face emotionally heavy situations. Severe injuries, anxiety, death, physical aggression and psychological aggression can lead to emotion exhaustion and depersonalization [1, 35], in residents [15, 21, 31].

# Lack of job resources

## Autonomy and participation

Freedom in decision making, based on knowledge and skills, is a protective factor against burnout in employees [36]. Participation in the decision process (both on the patient level, the department level and the organizational level) can lower the level of emotional exhaustion and can increase the level of depersonalization [37]. The position of the resident is characterized by a lot of responsibility and little experience [38], little autonomy and participation opportunities [25, 29]. Residents participating actively in the clinical decision making, controlling their own agenda or being heard about the content of the training program are less prone to develop burnout [19, 20, 25, 29, 31].

## Professional development

Professional development in employees can be established by the job content and by the opportunity to receive additional training [33]. Research opportunities, challenges at work, attending seminaries or workshops, and working with new technologies are examples to strengthen the professional development of a resident [39]. Professional development leads to less emotional exhaustion and less depersonalization [33].

## Feedback

Feedback is a powerful tool in the prevention of burnout in residents [12, 17, 24, 40], impacting the emotional exhaustion, feelings of depersonalization and feelings of personal accomplishment [34, 41]. Residents mainly receive feedback from their supervisor by supervision. Supervision creates a unique learning experience, in which the professional identity gets formed and professional skills will develop [42]. Supervisors can teach residents about the importance of breaks, rest, a balanced life and the delegation of tasks [38].

## Social support

Support of colleagues and supervisors is a pivotal aspect in the prevention of burnout [43], with an impact on emotional exhaustion, depersonalization and feelings of personal accomplishment [44-46]. The literature emphasizes the crucial role of supervisors [33].

## Organizational culture

Watts et al. [47] suggest that an innovative organization culture improves personal accomplishment in employees. An innovative organization does not mean revolutionary changes must take place. Small, incremental innovations can lead to big changes [47]. In addition, another publication found a positive effect of the innovative organization on emotional exhaustion [4].

## Communication and collaboration

A productive multidisciplinary working relationship decreases depersonalization and emotional exhaustion. Furthermore, good top-down communication leads to less emotional exhaustion [46, 48, 49]. Asymmetrical reciprocity between on the one hand residents and on the other hand nurses or supervisors increases the risk to develop burnout [50].

# References

[1] Alexander DA, Klein S. *Ambulance personnel and critical incidents: impact of accident and emergency work on mental health and emotional well-being*. The British journal of psychiatry : the journal of mental science 2001;**178**:76-81.

[2] Vlerick P. Onderzoek naar de antecedenten en gevolgen van burnout bij verpleegkundigen in algemene ziekenhuizen [Research of the antecedents and consequenses of burnout in nurses employed in general hospitals]. In: University G, (ed). Ghent, 1994:410.

[3] Maslach C, Schaufeli WB, Leiter MP. *Job burnout*. Annual Review of Psychology 2001;**52**:397-422.

[4] Adali E, & Priami, M. *Burnout among nurses in intensive care units, internal medicine wards and emergency departments in Greek hospitals*. Nurs Web J 2002;**11**:1-19.

[5] Jovanovic N, Podlesek A, Volpe U, Barrett E, Ferrari S, Rojnic Kuzman M *et al.* *Burnout syndrome among psychiatric trainees in 22 countries: Risk increased by long working hours, lack of supervision, and psychiatry not being first career choice*. European psychiatry : the journal of the Association of European Psychiatrists 2016;**32**:34-41.

[6] Van Dievoort N, Bronselaer, G., & Hoebeke, P. Onderzoek naar burnout in een universitair ziekenhuis [Burnout research in a university hospital]. Ghent University Psychiatry and Medical Psychology 2015.

[7] Adriaenssens J, De Gucht V, Maes S. *Determinants and prevalence of burnout in emergency nurses: a systematic review of 25 years of research*. International journal of nursing studies 2015;**52**:649-61.

[8] Goehring C, Bouvier Gallacchi M, Kunzi B, Bovier P. *Psychosocial and professional characteristics of burnout in Swiss primary care practitioners: a cross-sectional survey*. Swiss medical weekly 2005;**135**:101-8.

[9] Sanchez S, Mahmoudi R, Moronne I, Camonin D, Novella JL. *Burnout in the field of geriatric medicine: Review of the literature*. European Geriatric Medicine 2015;**6**:175-83.

[10] Prins JT, Gazendam-Donofrio SM, Tubben BJ, van der Heijden FM, van de Wiel HB, Hoekstra-Weebers JE. *Burnout in medical residents: a review*. Medical education 2007;**41**:788-800.

[11] Prins JT, Hoekstra-Weebers JE, van de Wiel HB, Gazendam-Donofrio SM, Sprangers F, Jaspers FC *et al.* *Burnout among Dutch medical residents*. International journal of behavioral medicine 2007;**14**:119-25.

[12] Galam E, Komly V, Le Tourneur A, Jund J. *Burnout among French GPs in training: a cross-sectional study*. The British journal of general practice : the journal of the Royal College of General Practitioners 2013;**63**:e217-24.

[13] Prins JT, Hoekstra-Weebers JE, Gazendam-Donofrio SM, Dillingh GS, Bakker AB, Huisman M *et al.* *Burnout and engagement among resident doctors in the Netherlands: a national study*. Medical education 2010;**44**:236-47.

[14] Frost RO, Marten P, Lahart C, Rosenblate R. *THE DIMENSIONS OF PERFECTIONISM*. Cognitive Therapy and Research 1990;**14**:449-68.

[15] Lue BH, Chen HJ, Wang CW, Cheng Y, Chen MC. *Stress, personal characteristics and burnout among first postgraduate year residents: a nationwide study in Taiwan*. Medical teacher 2010;**32**:400-7.

[16] Eley DS, Cloninger CR, Walters L, Laurence C, Synnott R, Wilkinson D. *The relationship between resilience and personality traits in doctors: implications for enhancing well being*. PeerJ 2013;**1**:e216.

[17] Ripp J, Babyatsky M, Fallar R, Bazari H, Bellini L, Kapadia C *et al.* *The incidence and predictors of job burnout in first-year internal medicine residents: a five-institution study*. Academic medicine : journal of the Association of American Medical Colleges 2011;**86**:1304-10.

[18] Schaufeli WB, Bakker AB, van der Heijden F, Prins JT. *Workaholism, burnout and well-being among junior doctors: The mediating role of role conflict*. Work and Stress 2009;**23**:155-72.

[19] Eckleberry-Hunt J, Lick D, Boura J, Hunt R, Balasubramaniam M, Mulhem E *et al.* *An exploratory study of resident burnout and wellness*. Academic medicine : journal of the Association of American Medical Colleges 2009;**84**:269-77.

[20] Bragard I, Etienne AM, Libert Y, Merckaert I, Lienard A, Meunier J *et al.* *Predictors and correlates of burnout in residents working with cancer patients*. Journal of cancer education : the official journal of the American Association for Cancer Education 2010;**25**:120-6.

[21] Panagopoulou E, Montgomery A, Benos A. *Burnout in internal medicine physicians: Differences between residents and specialists*. European journal of internal medicine 2006;**17**:195-200.

[22] Maslach C. *Job burnout: New directions in research and intervention*. Current Directions in Psychological Science 2003;**12**:189-92.

[23] Aydemir O, & Icelli, I. Burnout: risk factors. In: Bachler-Kohler, (ed). *Burnout for experts*. NY, Dordrecht, Heidelberg, London: Springer, 2013:119-45.

[24] Castelo-Branco C, Figueras F, Eixarch E, Quereda F, Cancelo MJ, Gonzalez S *et al.* *Stress symptoms and burnout in obstetric and gynaecology residents*. BJOG : an international journal of obstetrics and gynaecology 2007;**114**:94-8.

[25] Golub JS, Weiss PS, Ramesh AK, Ossoff RH, Johns MM, 3rd. *Burnout in residents of otolaryngology-head and neck surgery: a national inquiry into the health of residency training*. Academic medicine : journal of the Association of American Medical Colleges 2007;**82**:596-601.

[26] de Oliveira GS, Jr., Chang R, Fitzgerald PC, Almeida MD, Castro-Alves LS, Ahmad S *et al.* *The prevalence of burnout and depression and their association with adherence to safety and practice standards: a survey of United States anesthesiology trainees*. Anesthesia and analgesia 2013;**117**:182-93.

[27] Lindwall M, Ljung, T., Hadzibajramovic, E., & Jonsdottir, I.H. *Self-reported physical activity and aerobic fitness are differently related to mental health* Mental Health and Physical Activity 2012;**5**:28-34.

[28] Smith MJ, Conway FT, Karsh BT. *Occupational stress in human computer interaction*. Industrial health 1999;**37**:157-73.

[29] Ringrose R, Houterman S, Koops W, Oei G. *Burnout in medical residents: a questionnaire and interview study*. Psychology, health & medicine 2009;**14**:476-86.

[30] Dyrbye L, Shanafelt T. *A narrative review on burnout experienced by medical students and residents*. Medical education 2016;**50**:132-49.

[31] Blanchard P, Truchot D, Albiges-Sauvin L, Dewas S, Pointreau Y, Rodrigues M *et al.* *Prevalence and causes of burnout amongst oncology residents: a comprehensive nationwide cross-sectional study*. European journal of cancer (Oxford, England : 1990) 2010;**46**:2708-15.

[32] Landrigan CP, Fahrenkopf AM, Lewin D, Sharek PJ, Barger LK, Eisner M *et al.* *Effects of the accreditation council for graduate medical education duty hour limits on sleep, work hours, and safety*. Pediatrics 2008;**122**:250-8.

[33] Schaufeli WB, & Enzmann, D. *The burnout companion to study and practice: a critical analysis.* London: Taylor and Francis, 1998.

[34] Vanbelle E, De Witte, H., Moerenhout, E., Vandenbroeck, S., Vanhaecht, K., & Godderis, L. Burn-out in de zorg: afbakening en overzicht van oorzaken en gevolgen [burnout in the health care sector: demarcation and overview of causes and consequenses]. In: Van der Borght M, (ed). *HR in de zorg*. Brussel: Uitgeverij Politeia NV, 2013.

[35] Bernaldo-De-Quiros M, Piccini AT, Gomez MM, Cerdeira JC. *Psychological consequences of aggression in pre-hospital emergency care: cross sectional survey*. International journal of nursing studies 2015;**52**:260-70.

[36] Jourdain G, Chenevert D. *Job demands-resources, burnout and intention to leave the nursing profession: a questionnaire survey*. International journal of nursing studies 2010;**47**:709-22.

[37] Laschinger HKS, Fida R. *A time-lagged analysis of the effect of authentic leadership on workplace bullying, burnout, and occupational turnover intentions*. European Journal of Work and Organizational Psychology 2014;**23**:739-53.

[38] Schaufeli WB. Burnout. In: Firth-Cozens J, Payne, R.,, (ed). *Stress in health professionals: psychological and organizational causes and interventions*. Chichester, NY: Wiley, 1999.

[39] Zis P, Artemiadis AK, Lykouri M, Xirou S, Roussopoulou A, Papageorgiou E *et al.* *Residency Training: Determinants of burnout of neurology trainees in Attica, Greece*. Neurology 2015;**85**:e81-4.

[40] Chaput B, Bertheuil N, Jacques J, Smilevitch D, Bekara F, Soler P *et al.* *Professional Burnout Among Plastic Surgery Residents Can it be Prevented? Outcomes of a National Survey*. Ann Plast Surg 2015;**75**:2-8.

[41] Thomas CH, Lankau MJ. *Preventing Burnout: The Effects of LMX and Mentoring on Socialization, Role Stress, and Burnout*. Human Resource Management 2009;**48**:417-32.

[42] Falender CA, & Shafranske, E.P. *Clinical supervision: a competency-based approach*. Washington, DC: American Psychological Association, 2004.

[43] Vlerick P, & Coetsier, P. Onderzoek naar de releatie tussen de werk-psychologische antecedenten werkstress, ondersteuning en burnout [inquiry of the relationship between the work-psychological antecedents workstress, social support and burnout]. In: Bouwen R, De Witte, K, & Verboven, J., (ed). *Organiseren en veranderen [Organizing and changing]*. Leuven: Garant, 1996:339-63.

[44] Garcia-Izquierdo M, Rios-Risquez MI. *The relationship between psychosocial job stress and burnout in emergency departments: an exploratory study*. Nursing outlook 2012;**60**:322-9.

[45] Moors K, Symons, C., Van Der Meeren, E., De Witte, K., & Evers, G. Burnout bij Vlaamse verpleegkundigen [burnout in Flemish nurses]. 2001

[46] Van Der Ploeg E, & Kleber, R. *Ingrijpende gebeurtenissen op het werk en chronische stressoren [Major Events at Work and Chronic Stressors]*. Gedrag Organ 2001;**14**:124-51.

[47] Watts J, Robertson N, Winter R, Leeson D. *Evaluation of organisational culture and nurse burnout*. Nursing management (Harrow, London, England : 1994) 2013;**20**:24-9.

[48] O'Mahony N. *Nurse burnout and the working environment*. Emergency nurse : the journal of the RCN Accident and Emergency Nursing Association 2011;**19**:30-7.

[49] Spence Laschinger HK, Leiter MP. *The impact of nursing work environments on patient safety outcomes: the mediating role of burnout/engagement*. The Journal of nursing administration 2006;**36**:259-67.

[50] Prins JT, Gazendam-Donofrio SM, Dillingh GS, van de Wiel HB, van der Heijden FM, Hoekstra-Weebers JE. *The relationship between reciprocity and burnout in Dutch medical residents*. Medical education 2008;**42**:721-8.
